# Editorial: Exploiting biomarkers for targeted therapies in acute myeloid leukemia

**DOI:** 10.3389/fmed.2026.1813324

**Published:** 2026-03-12

**Authors:** Diego Carbonell

**Affiliations:** 1 Department of Hematology, Gregorio Marañón General University Hospital, Madrid, Spain; 2 Gregorio Marañón Health Research Institute (IiSGM), Madrid, Spain

**Keywords:** acute myeloid leukemia, biomarkers, cell therapy, chemotherapy resistance, immunotherapy, precision medicine, therapeutic targets

Acute myeloid leukemia (AML) remains a biologically complex and clinically aggressive hematologic malignancy. Rather than representing a single disease entity, AML encompasses a spectrum of biologically diverse conditions characterized by genetic complexity, epigenetic heterogeneity, and marked cellular plasticity (1, 2). Its clinical course reflects not only intrinsic molecular alterations but also dynamic interactions between leukemic cells, the bone marrow niche, and the selective pressures imposed by therapy (3–5). Although genomic characterization has advanced considerably and multiple targeted agents have entered clinical practice, durable disease control is still achieved in only a subset of patients (6). The persistence of relapse reflects the adaptive capacity of leukemic cells and exposes the limits of static molecular classifications. In this evolving landscape, biomarker research has progressively shifted from simple categorization toward a more functional understanding of disease behavior (7–9). Against this background, this Research Topic, “*Exploiting Biomarkers for Targeted Therapies in Acute Myeloid Leukemia*,” brings together contributions that explore these dimensions from complementary perspectives, spanning molecular targeting, resistance biology, transcriptomic subtyping, and hypoxia-driven disease mechanisms.

In practice, this conceptual shift has translated into a growing effort to define signaling dependencies that may be therapeutically exploited. Kuang et al. examine this approach through the investigation of a novel 3,3′-diindolylmethane derivative (L1) in erythroleukemia models. Their study demonstrates that L1 induces apoptosis via activation of endoplasmic reticulum stress pathways while concurrently inhibiting the FLI1/AKT signaling axis. Beyond documenting anti-proliferative effects, the authors provide mechanistic insight into molecular mediators associated with drug response, including HSP70 and FLI1. By linking pathway modulation to defined cellular outcomes, the study illustrates how functional analyses can complement genomic characterization and help identify candidate biomarkers of therapeutic sensitivity. Such work contributes to a more biologically grounded framework for interpreting treatment response in AML.

Therapeutic resistance remains a central challenge in AML management. Although recurrent genetic alterations contribute to treatment failure, mutational status alone does not fully account for disease persistence following therapy. Increasing attention has therefore turned to non-genetic and potentially reversible adaptations that allow leukemic cells to withstand pharmacologic stress (10). Li et al. provide a comprehensive overview of drug-tolerant persister (DTP) cells in AML, describing a subpopulation capable of surviving therapy through mechanisms such as metabolic reprogramming, chromatin remodeling, and transient quiescence. These adaptive states are dynamic and reversible, enabling leukemic expansion once treatment pressure diminishes. In this context, DTP cells represent a plausible reservoir for minimal residual disease and subsequent relapse. Recognizing these fluctuating cellular states broadens the scope of biomarker research beyond static molecular alterations. This underscores the need for longitudinal approaches and functional assays capable of capturing adaptive shifts over time, particularly with targeted therapies.

The bone marrow microenvironment adds another layer of influence on leukemic behavior and treatment response (11). Among its defining features, hypoxia plays a significant role in shaping transcriptional programs and metabolic pathways within AML cells. Liu et al. investigate hypoxia-related gene signatures and their clinical relevance, demonstrating associations with immune microenvironment alterations, modulation of mTOR signaling, and features consistent with drug resistance. By constructing a prognostic model based on hypoxia-associated genes, the authors illustrate how microenvironment-linked transcriptional programs may contribute to patient stratification and risk assessment. These findings reinforce the importance of considering extrinsic regulatory signals when interpreting leukemic biology. AML progression and therapeutic response are therefore shaped not only by cell-intrinsic alterations, but also by contextual cues within the marrow niche.

Given the marked heterogeneity of AML, refining stratification strategies remains an ongoing priority. While mutation-based classifications have significantly improved prognostic assessment, they may not fully capture coordinated biological programs operating within leukemic cells. Zhong et al. address this limitation through a pathway enrichment–based subtyping framework derived from transcriptomic analysis. They identify three AML subtypes characterized by differential activation of DNA repair, immune, metabolic, and oncogenic pathways. These subtypes show distinct stemness features, proliferative capacity, immune signatures, and predicted drug sensitivities. By focusing on pathway-level activity rather than individual gene mutations, this approach offers a more integrated perspective on leukemic biology. It suggests that coordinated signaling programs may provide complementary information for therapeutic alignment and patient stratification, particularly as treatment options become increasingly diverse.

Taken together, the contributions in this Research Topic converge on a shared observation: AML biology cannot be adequately interpreted through isolated molecular findings. Instead, therapeutic response and disease progression emerge from the interplay between signaling dependencies, adaptive cellular states, and microenvironmental influences. The mechanistic interrogation of actionable pathways, the recognition of drug-tolerant persister states, and the impact of hypoxia-driven transcriptional programs collectively support a more contextual understanding of the disease. Pathway-based subtyping represents an attempt to integrate these layers within clinically interpretable frameworks. Static molecular descriptors remain essential, but they require functional and temporal context if they are to inform therapeutic decisions in a meaningful way. Progress in this area will likely depend on longitudinal sampling strategies and multi-omic integration capable of capturing biological evolution under therapeutic pressure (12). Early identification of adaptive states may help refine combination approaches and limit the emergence of resistance. At the same time, biomarker discovery must be accompanied by careful clinical validation to ensure reproducibility and applicability in routine practice. As treatment strategies continue to diversify, the need for biomarker frameworks that reflect disease dynamics rather than fixed classifications becomes increasingly evident. The studies assembled here contribute to this evolving perspective and highlight the importance of integrating mechanistic insight with clinical relevance in the ongoing effort to improve outcomes in AML.
